# A Novel Scoring System Based on Preoperative Routine Blood Test in Predicting Prognosis of Atypical Meningioma

**DOI:** 10.3389/fonc.2020.01705

**Published:** 2020-09-04

**Authors:** Xiaoyong Chen, Guojun Wang, Jianhe Zhang, Gaoqi Zhang, Yuanxiang Lin, Zhangya Lin, Jianjun Gu, Dezhi Kang, Chenyu Ding

**Affiliations:** ^1^Department of Neurosurgery, The First Affiliated Hospital of Fujian Medical University, Fuzhou, China; ^2^Department of Neurosurgery, Binhai County People’s Hospital, Yancheng, China; ^3^Department of Neurosurgery, The Affiliated Hospital of Putian University, Putian, China; ^4^Department of Neurosurgery, Zhengzhou University People’s Hospital, Henan Provincial People’s Hospital, Zhengzhou, China; ^5^Fujian Provincial Key Laboratory of Precision Medicine for Cancer, Fuzhou, China

**Keywords:** atypical meningioma, fibrinogen, neutrophil-lymphocyte ratio, progression-free survival, prognosis

## Abstract

**Purpose:**

The aim of this study was to explore the correlation and clinical significance of preoperative fibrinogen and neutrophil-lymphocyte ratio (F-NLR) scoring system with 3-year progression-free survival (PFS) of patients with atypical meningioma.

**Materials and Methods:**

Clinical, pathological, radiological, and laboratory variables were collected to analyze their correlation with 3-year PFS in the training set with 163 patients. Patients were classified by different F-NLR scores (0, 1, or 2). External validation for the predictive value of F-NLR scoring system was performed in the validation set with 105 patients.

**Results:**

Overall, 37.3% (100 of 268) of the enrolled patients were male. The scoring system showed good performance in predicting 3-year PFS (AUC = 0.872, 95%CI = 0.811–0.919, sensitivity = 66.1%, specificity = 93.3%, and *Youden* index = 0.594). DeLong’s test indicated that the AUC of F-NLR scoring system was significantly greater than that of fibrinogen level and NLR (*Z* = 2.929, *P* = 0.003; *Z* = 3.376, *P* < 0.001). Multivariate Cox analysis revealed that tumor size (HR = 1.39, 95%CI = 1.10–1.76, *P* = 0.007), tumor location (HR = 3.11, 95%CI = 1.60–6.95, *P* = 0.001), and F-NLR score (score of 1: HR = 12.78, 95%CI = 3.78–43.08, *P* < 0.001; score of 2: HR = 44.58, 95%CI = 13.02–152.65, *P* < 0.001) remained significantly associated with 3-year PFS. The good predictive performance of F-NLR scoring system was also demonstrated in the validation set (AUC = 0.824, 95%CI = 0.738–0.891, sensitivity = 62.5%, specificity = 87.9%, and *Youden* index = 0.504).

**Conclusion:**

Our study confirmed the correlation and clinical significance of preoperative F-NLR scoring system with 3-year PFS of patients with atypical meningioma. A prospective and large-scale study is required to validate our findings.

## Introduction

Meningioma is a common type of intracranial tumor with three grades of malignancy (1). Atypical meningioma is considered as a transitional type between benign and malignant meningioma. To some extent, the WHO grade II meningioma presents a malignant tendency with an approximately recurrence rate of 40% (2, 3). Patients with atypical meningioma had an average rate of 50% for 5-year progression-free survival (PFS) (4). The optimal management of patients with atypical meningioma remained controversial mainly because of the difficulty for predicting tumor recurrence. The existing studies suggested that surgical resection, imaging, postoperative radiation therapy (PORT), and pathologic features could be reliable predictors for atypical meningioma (5–8). However, there is no general consensus on which predicative factor is most clinically effective and meaningful.

The important roles of systemic inflammatory response and coagulation cascade have been confirmed (9). Recently, studies have emphasized that hyperfibrinogenemia is related to the malignant behaviors and poor prognosis in various types of tumor (10–13). Inflammatory biomarkers like neutrophil-lymphocyte ratio (NLR) in peripheral blood could reflect the state of body inflammatory response and have been confirmed to be related with recurrence in many malignancies (14–17). However, the clinical significance of plasma level of fibrinogen and NLR has not been clarified for patients with atypical meningiomas.

The present study is aimed to explore the correlation and clinical significance of preoperative F-NLR scoring system with 3-year PFS of patients with atypical meningioma.

## Materials and Methods

### Study Population

This study was performed at the Department of Neurosurgery of The First Affiliated Hospital of Fujian Medical University, the Department of Neurosurgery of The Affiliated Hospital of Putian University and the Department of Neurosurgery of Zhengzhou University People’s Hospital. It was approved by local Ethics Committee of all participating hospitals and conformed to the Ethical guidelines of the Declaration of Helsinki. The requirement of informed consent was waived due to its retrospective design. Patients with atypical meningioma admitted to the three institutions between January 2007 and January 2017 were enrolled in this retrospective observational study. Possible related factors for 3-year PFS were identified on data from 163 patients operated between January 2007 and December 2013 (training set). External validation was performed with data from 105 patients operated from January 2014 to January 2017 (validation set). The cohort consisted of 268 patients meeting the following inclusion criteria: (1) age >18 years old; (2)a preoperative diagnosis of meningioma based on imaging analysis and a confirmed diagnosis of atypical meningioma based on pathological results; (3) peripheral blood test was performed within 7 days before surgery. Exclusion criteria were: (1) incomplete medical information such as missing the peripheral blood test; (2) history of other tumors; (3) patients had previous atypical meningioma surgery or other surgery; (4) evidence of infection or previous use of steroids, antitumor drugs, antiplatelet or anticoagulant drugs, or immunosuppressants; (5) combined with other neurological diseases or systemic diseases.

### Pathological Examination

All patients met the diagnostic criteria of atypical meningioma. The 2007 WHO histological criteria included (18): (1) three or more of the five histological features: high cellularity, geographic necrosis, nuclear pleomorphism, foci of small hyperchromatic cells, and uninterrupted pattern-less or sheet-like growth; (2) a mitotic index of four or higher per ten high-power fields (HPF). Brain invasion was supplemented as an additional criterion in the 2016 WHO edition (1). We extracted the value of mitotic index per ten HPF, Ki-67 index, and presence or absent of brain invasion for all patients.

### Radiological Examination

For each case, preoperative magnetic resonance imaging (MRI) or computed tomography (CT) scan was performed to evaluate the site and diameter of tumor. Tumor site was classified into skull base group and non-skull base group (19). Tumor originating from cavernous sinus, anterior clinoid, tuberculum sella, optic sheath, planum sphenoidale, medial tentorial, petroclival, foramen magnum, bony foramina, lateral/middle sphenoid wing, posterior petrous, orbital roof, and lateral tentorial were assigned into skull base group. Non-skull base group contained tumor arising from convexity, parasagittal, falcine, cerebellar convexity, intraventricular, and pineal. Medical records and postoperative imaging data within 1 month after surgery were retrieved to classify the extent of surgical resection based on Simpson’s scale (20). Because of the insufficient samples in the Simpson grade IV and Simpson grade V, all patients were divided into Simpson grade I-II group and Simpson grade III-V group to distinguish the extent of surgical excision for the further analysis. Except for the above-mentioned characteristics, two neuroradiologists blinded to the medical data also independently analyzed the preoperative and postoperative MRI or CT images to determine the extent of peritumoral edema.

### Clinical and Laboratory Variables

Patient data including history of present illness, past medical history, general demographics, treatment regimens (PORT), and other related data were collected. All patients underwent general preoperative blood tests including routine blood examinations according to standard laboratory test procedures within 1 week before surgery. The normal values for fibrinogen range from 1.8 to 3.5 g/L. Based on the cut-off value, we classified the F-NLR scores as 0[neither hyperfibrinogenemia (fibrinogen-lymphocyte ratio >2.95) nor high NLR(>2.74)], 1[hyperfibrinogenemia or high NLR], or 2[both hyperfibrinogenemia and high NLR].

### Follow-Up Evaluation

All patients underwent contrast-enhanced CT scan or MR imaging within 1 month after surgery. The subsequent intervals of follow-up ranged from 3 to 6 months. Progression-free survival in this manuscript was defined as the time from surgery to relapse. At the end point, the 3-year PFS of patients after surgery were estimated. Patients with growing residual tumor or new lesions on a follow up contrast-enhanced MR imaging or CT were included into the recurrence group. The other patients were included into the non-recurrence group.

### Statistical Analysis

Statistical analysis was performed using SPSS 17.0 software (SPSS, Inc., Chicago, Illinois, United States). Continuous variables, described as mean ± standard deviation, were analyzed using 2-sample *t-*test. Categorical variables, expressed as counts (percentage), were analyzed using Pearson χ^2^ test or Fisher exact test. All available variables were included in univariate logistic regression analysis for their association with 3-year PFS. Variables which had univariate association of *P* < 0.10 were included for further multivariate analysis. Backward stepwise multivariate regression was performed to create the final model of which the variables had *P* < 0.05. The predictive value of variables was assessed by the receiver operating characteristic (ROC) curve analysis. The best threshold of predictor was determined with its sensitivity, specificity, and *Youden* index. DeLong’s test was used to assess the model performance of area under the curve (AUC). Kaplan-Meier curve analysis was performed to evaluate 3-year PFS rate after surgery. *P*-value for comparing survival curves was calculated with the log-rank test. The univariate and multivariate Cox proportional models were utilized to assess the prognostic significance of included factors. A value less than 0.05 was considered statistically significant.

## Results

### Patient Characteristics

In this study, atypical meningioma patients were divided into a recurrence group (*n* = 78, 29.10%) and a non-recurrence group (*n* = 190, 70.90%). Overall, the average age of patients was 54.00 ± 11.69 years old; 37.3% (100 of 268) of the enrolled patients were male and 62.7% of them were female.

The entire cohort including 268 patients was divided into the training set and the validation set. Forty-five cases of recurrence were observed in 163 patients of the training set (27.6%) and thirty-three cases were observed in the 105 patients of the validation set (31.4%).

Table 1 shows the demographics and baseline characteristics of the 163 patients in the training set. Age, neutrophil count, lymphocyte count, NLR, plasma fibrinogen level, tumor size, and extent of resection were significantly different between the two groups. The difference of tumor location for the two 3-year PFS rate groups was not significant with *P* = 0.082, so did the PORT with *P* = 0.095.

**TABLE 1:** Comparison of demographic and clinical variables in patients with PFS < 3 and PFS ≥ 3 in the training set.

Parameter	Total	PFS < 3 year (*n* = 45)	PFS ≥ 3 year (*n* = 118)	*P*-value
Age	54.45 ± 11.68	57.87 ± 11.13	53.15 ± 11.67	0.021
Sex				0.404
Male	59 (36.2%)	14 (31.1%)	45 (38.1%)	
Female	104 (63.8%)	31 (68.9%)	73 (61.9%)	
Hypertension				0.649
No	134 (82.2%)	36 (80.0%)	99 (83.1%)	
Yes	29 (17.8%)	9 (20.0%)	20 (16.9%)	
Diabetes mellitus				0.566
No	155 (95.1%)	44 (97.8%)	111 (94.1%)	
Yes	8 (4.9%)	1 (2.2%)	7 (5.9%)	
WBC count 10∧9/L	6.57 ± 2.29	6.92 ± 2.39	6.44 ± 2.26	0.238
NEU count 10∧9/L	4.25 ± 2.16	4.99 ± 2.31	3.97 ± 2.03	0.007
RBC count 10∧12/L	4.53 ± 0.57	4.57 ± 0.49	4.52 ± 0.60	0.585
MON count 10∧9/L	0.40 ± 0.43	0.32 ± 0.26	0.43 ± 0.48	0.178
LYM count 10∧9/L	1.83 ± 0.55	1.64 ± 0.48	1.91 ± 0.56	0.004
PLT count 10∧9/L	239.77 ± 82.33	246.33 ± 70.55	237.28 ± 86.55	0.532
HGB g/L	133.75 ± 15.63	133.06 ± 14.42	134.01 ± 16.12	0.730
FIB g/L	2.99 ± 0.91	3.61 ± 1.16	2.73 ± 0.65	< 0.001
NLR	2.55 ± 1.70	3.24 ± 1.39	2.29 ± 1.75	0.001
Tumor size cm	4.93 ± 1.44	5.55 ± 1.34	4.69 ± 1.41	0.001
Tumor location				0.082
Non-skull base	124 (76.1%)	30 (66.7%)	94 (79.7%)	
Skull base	39 (23.9%)	15 (33.3%)	24 (20.3%)	
Extent of resection				0.012
Simpson Grade I-II	141 (86.5%)	34 (75.6%)	107 (90.7%)	
Simpson Grade III-V	22 (13.5%)	11 (24.4%)	11 (9.3%)	
Skull invasion				0.321
No	97 (59.5%)	24 (53.3%)	73 (61.9%)	
Yes	66 (40.5%)	21 (46.7%)	45 (38.1%)	
Peritumoral edema				0.321
Mild (≤1 cm)	66 (40.5%)	21 (46.7%)	45 (38.1%)	
Severe (>1 cm)	97 (59.5%)	24 (53.3%)	73 (61.9%)	
Brain invasion				0.692
No	72 (44.2%)	21 (46.7%)	51 (43.2%)	
Yes	91 (55.8%)	24 (53.3%)	67 (56.8%)	
Mitotic level				0.132
<4/HPF	102 (62.6%)	24 (53.3%)	78 (66.1%)	
≥4/HPF	61 (37.4%)	21 (46.7%)	40 (33.9%)	
Ki-67 index				0.563
<5	100 (61.3%)	26 (57.8%)	74 (62.7%)	
≥5	63 (38.7%)	19 (42.2%)	44 (37.3%)	
PORT				0.095
No	136 (83.4%)	34 (75.6%)	102 (86.4%)	
Yes	27 (16.6%)	11 (24.4%)	16 (13.6%)	

*Values are reported as number, number(%), and mean ± standard deviation. PFS, progression-free survival; WBC, white blood cell; NEU, neutrophil; RBC, red blood cell; MON, monocyte; LYM, lymphocyte; PLT, platelet; HGB, hemoglobin; FIB, fibrinogen; NLR, neutrophil-lymphocyte ratio; PORT, postoperative radiation therapy.*

### Univariate and Multivariate Analysis of the Factors Related With 3-Year PFS

Parameters with significant univariate association (*P* < 0.10) for 3-year PFS were shown in Table 2, including age, NLR, plasma fibrinogen level, tumor size, tumor location, extent of resection, and PORT. After multivariate analysis, NLR (OR = 0.77, 95%CI = 0.62–0.99, *P* = 0.025), and plasma fibrinogen level (OR = 0.27, 95%CI = 0.15–0.48, *P* < 0.001) were still significant after adjusting for confounders. In addition, tumor size (OR = 0.59, 95%CI = 0.43–0.81, *P* = 0.001), and extent of resection (OR = 3.43, 95%CI = 1.12-10.51, *P* = 0.031) remained significant and independent of 3-year PFS.

**TABLE 2:** Univariate and multivariate analysis of 3-year PFS with possible predictive factors in the training set.

Parameter	Univariate analysis			Multivariate analysis		
	OR	95%CI	*P*-value	OR	95%CI	*P*-value
Age	0.96	0.93–1.00	0.023			
NLR	0.73	0.59–0.91	0.004	0.77	0.61–0.99	0.025
FIB	0.26	0.15–0.45	<0.001	0.27	0.15–0.48	<0.001
Tumor size cm	0.64	0.49–0.84	0.001	0.59	0.43–0.81	0.001
Tumor location	0.85	0.23–1.10	0.085			
Extent of resection	3.15	1.25–7.90	0.015	3.43	1.12-10.51	0.031
PORT	2.06	0.87–4.88	0.099			

*OR, odds ratio; CI, confidence interval; NEU, neutrophil; LYM, lymphocyte; FIB, fibrinogen; NLR, neutrophil-lymphocyte ratio; PORT, postoperative radiation therapy.*

### Comparison of the Prognostic Value of Preoperative Plasma Biomarkers

**FIGURE 1:**
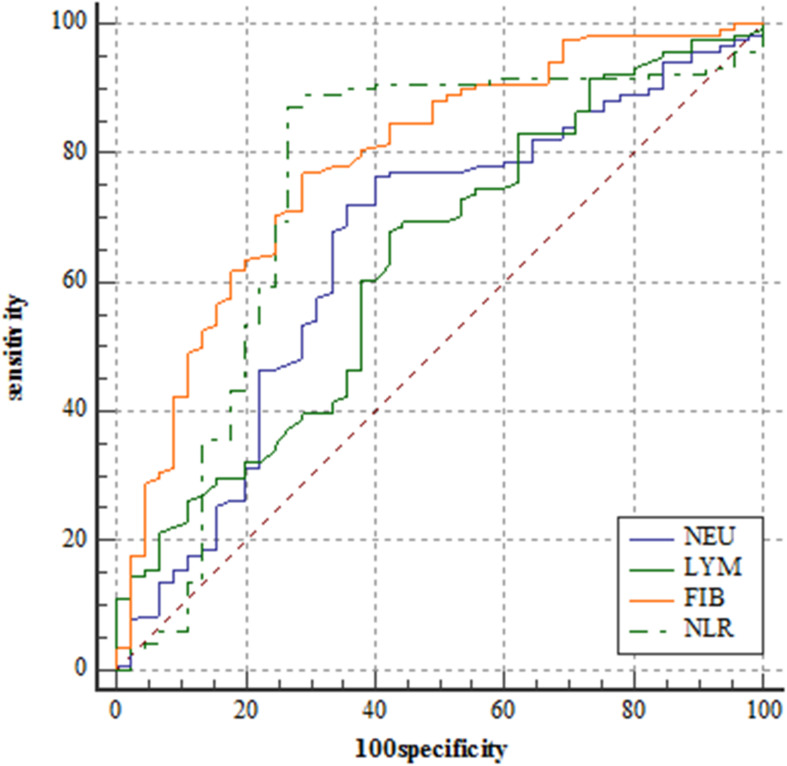
Receiver operating characteristic curve analyses comparing neutrophil counts, lymphocyte counts, fibrinogen level, and neutrophil-lymphocyte ratio for predicting patients reaching 3-year progression-free survival in the training set.

Utilizing ROC curve analysis, Figure 1 shows the prognostic value of the 4 preoperative plasma biomarkers. The best cut-off value of fibrinogen level for predicting 3-year PFS was 2.95 g/L. The predictive performance was represented with AUC = 0.786 (95%CI = 0.715–0.846), sensitivity = 77.1%, specificity = 71.1%, and *Youden* index = 0.482. Based on the best cut-off value of 4.00 × 10∧9/L, the predictive performance of neutrophil count (AUC = 0.652, 95%CI = 0.574–0.725, sensitivity = 72.0%, specificity = 64.4%, and *Youden* index = 0.365) was also calculated by ROC curve analysis (Figure 1). The best cut-off value of lymphocyte count for predicting 3-year PFS was 1.65 × 10∧9/L. The predictive performance was represented by AUC = 0.630 (95%CI = 0.551–0.704), sensitivity = 67.8%, specificity = 57.8%, and *Youden* index = 0.256. The cut-off value was 2.74 for NLR (AUC = 0.743, 95%CI = 0.669–0.808, sensitivity = 87.3%, specificity = 73.3%, and *Youden* index = 0.606).

DeLong’s test indicated that the AUC of fibrinogen level was comparable with that of NLR (*Z* = 0.711, *P* = 0.477). The AUC of NLR was significantly greater than that of neutrophil count (*Z* = 3.153, *P* = 0.002) and lymphocyte count (*Z* = 2.138, *P* = 0.033).

### Relationship Between F-NLR Score and Prognosis in the Training Set

**FIGURE 2:**
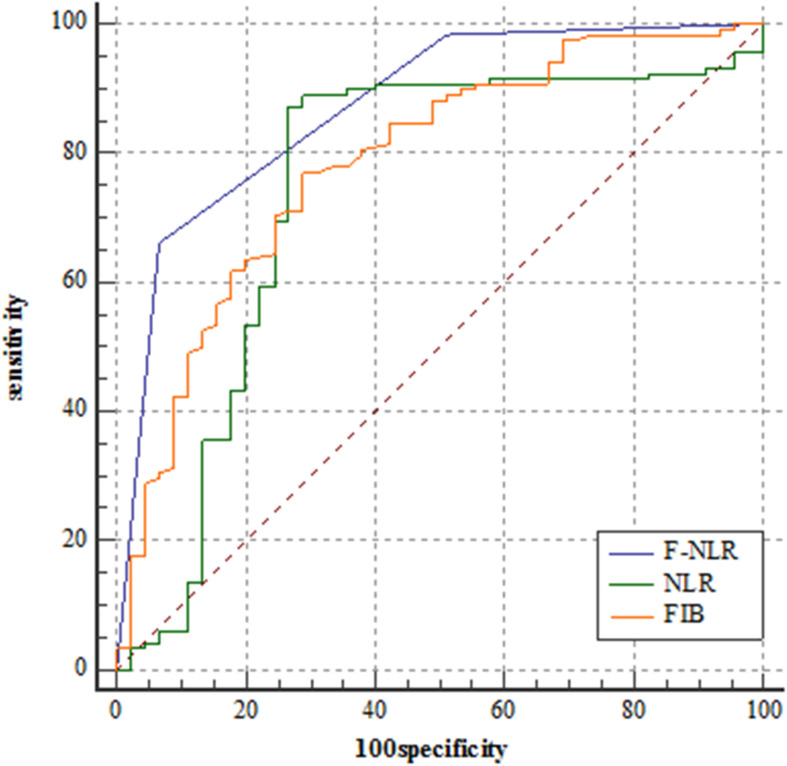
Receiver operating characteristic curve analyses comparing fibrinogen and neutrophil-lymphocyte ratio scoring system, fibrinogen level, and neutrophil-lymphocyte ratio for predicting patients reaching 3-year progression-free survival in the training set.

Figure 2 showed the predictive performance of F-NLR scoring system compared to fibrinogen level and NLR. Based on the cut-off value of 0, the scoring system had an AUC of 0.872 (95%CI = 0.811–0.919), a sensitivity of 66.1%, a specificity of 93.3%, and a *Youden* index of 0.594. DeLong’s test indicated that the AUC of F-NLR scoring system was significantly greater than those of fibrinogen level and NLR (*Z* = 2.929, *P* = 0.003; *Z* = 3.376, *P* < 0.001).

**FIGURE 3:**
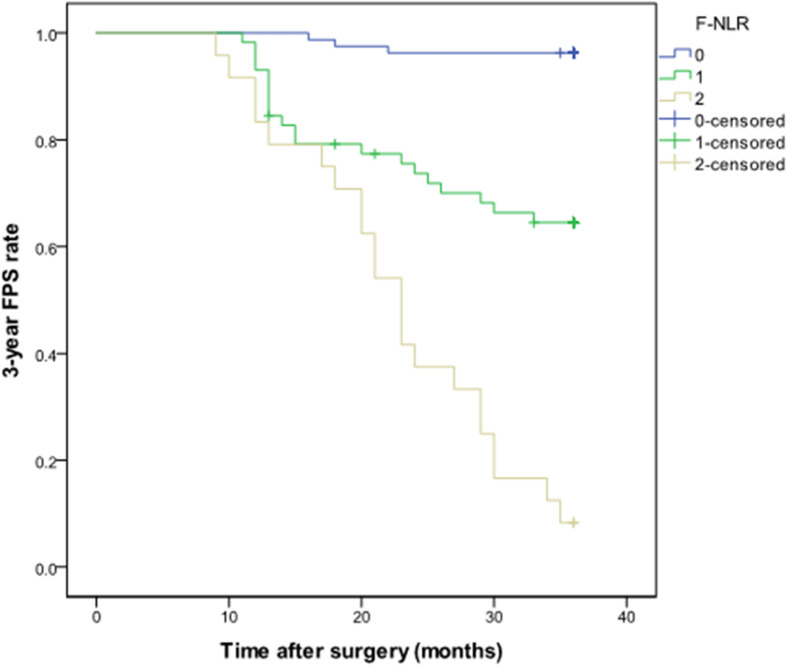
Kaplan-Meier curve of the 3-year progression-free survival rate in patients with different fibrinogen and neutrophil-lymphocyte ratio score from 0 to 2 in the training set.

The F-NLR scores were 0, 1, and 2 in 81 (49.7%), 58 (35.6%), and 24 (14.7%) of the 163 patients, respectively. The F-NLR score was significantly different in the patients with and without tumor recurrence at 3 years after surgery (*P* < 0.001). Patients with F-NLR score of 0–2 had significantly different 3-year PFS rate (96.3%, 78/81; 65.5%, 38/58; 8.3%, 2/24; *P* < 0.001, Figure 3). The mean 3-year PFS was 35.36 (95%CI = 34.64–36.08) months, 29.79 (95%CI = 27.36–32.22) months, 23.00 (95%CI = 19.75–26.50) months, respectively (*P* < 0.001, Figure 3).

In the univariate Cox hazard regression analysis, age (HR = 1.03, 95%CI = 1.00–1.06, *P* = 0.024), tumor size (HR = 1.46, 95%CI = 1.17–1.82, *P* = 0.001), tumor location (HR = 1.85, 95%CI = 0.99–3.43, *P* = 0.053), extent of resection (HR = 2.59, 95%CI = 1.31–5.12, *P* = 0.006), and F-NLR score (*P* < 0.001) were all significantly associated with 3-year PFS. Furthermore, multivariate Cox analysis revealed that tumor size (HR = 1.39, 95%CI = 1.10–1.76, *P* = 0.007), tumor location (HR = 3.11, 95%CI = 1.60–6.95, *P* = 0.001), and F-NLR score (score of 1: HR = 12.78, 95%CI = 3.78–43.08, *P* < 0.001; score of 2: HR = 44.58, 95%CI = 13.02–152.65, *P* < 0.001) remained associated with 3-year PFS (Table 3).

**TABLE 3:** Univariate and multivariate cox hazard regression analysis of 3-year PFS with possible predictive factors in the training set.

Parameter	Univariate analysis			Multivariate analysis		
	HR	95%CI	*P*-value	HR	95%CI	*P*-value
Age	1.03	1.00–1.06	0.024			
Tumor size cm	1.46	1.17–1.82	0.001	1.39	1.10–1.76	0.007
Tumor location	1.85	0.99–3.43	0.053	3.11	1.60–6.95	0.001
Extent of resection	2.59	1.31–5.12	0.006			
F-NLR score			<0.001			<0.001
0	1.00	Reference		1.00	Reference	
1	12.09	3.59–40.71	<0.001	12.78	3.78–43.08	<0.001
2	44.35	13.15–149.58	<0.001	44.58	13.02–152.65	<0.001

*HR, hazard ratio; CI, confidence interval; F-NLR, fibrinogen and neutrophil-lymphocyte ratio.*

### Relationship Between F-NLR Score and 3-Year PFS by Subgroup Analysis Based on Extent of Resection and Tumor Location

**FIGURE 4:**
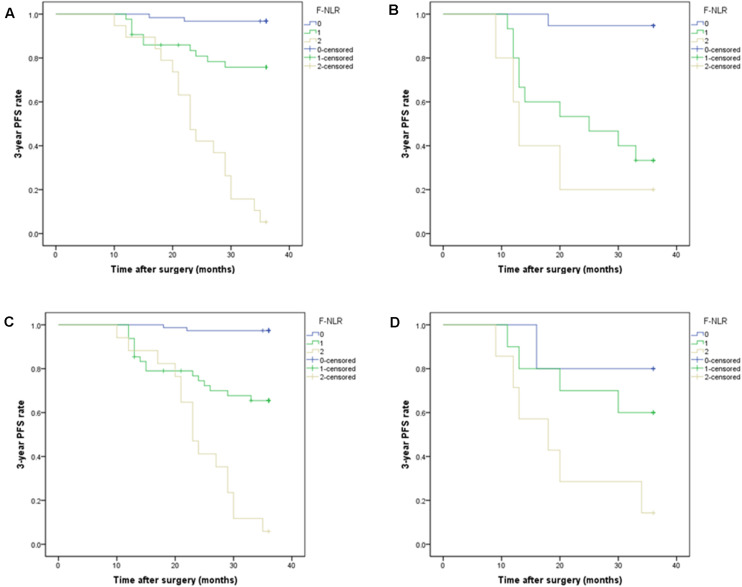
**(A,B)** Subgroup analysis of Kaplan-Meier curve of the 3-year progression-free survival rate in patients with different fibrinogen and neutrophil-lymphocyte ratio score from 0 to 2 based on tumor location in the training set. **(A)** F-NLR score was associated with 3-year PFS in non-skull base tumors, *P* < 0.001. **(B)** F-NLR score was associated with 3-year PFS in skull base tumors, *P* < 0.001. **(C,D)** Subgroup analysis of Kaplan-Meier curve of the 3-year PFS rate in patients with different F-NLR scores based on extent of resection. **(C)** F-NLR score was associated with 3-year PFS in patients with Simpson grade I-II resection, *P* < 0.001. **(D)** F-NLR score was not associated with 3-year PFS in patients with Simpson grade III-V resection, *P* = 0.045.

A significant interaction effect was found between extent of resection and F-NLR score, *P* < 0.001. In patients with Simpson grade I-II, Kaplan-Meier curves analysis revealed that patients with F-NLR score of 0–2 were significantly different in 3-year PFS rate (97.4%, 74/76; 66.7%, 32/48; 5.9%, 1/17) and mean 3-year PFS (35.58 months, 95%CI = 35.00–36.16; 29.95 months, 95%CI = 27.31–32.60; 24.12 months, 95%CI = 20.81–27.42) (*P* < 0.001, Figure 4). In patients receiving Simpson grade III-V resection, those with F-NLR score of 0–2 were not significantly different in 3-year PFS rate (80.0%, 4/5; 60.0%, 6/10; 14.3%, 1/7) and mean 3-year PFS (32.00 months, 95%CI = 24.99–39.01; 29.00 months, 95%CI = 22.94–35.06; 20.29 months, 95%CI = 12.93–27.64) (*P* = 0.045, Figure 4).

A significant interaction effect between tumor location and F-NLR score was also found for 3-year PFS, *P* < 0.001. In the Kaplan-Meier curves analysis of subgroups based on tumor location, F-NLR score was still associated with 3-year PFS (*P* < 0.001, Figure 4). For patients with non-skull base tumor, the 3-year PFS rates in F-NLR score 0–2 were 96.8% (60/62), 76.7% (33/43), and 5.3% (1/19), respectively; the mean 3-year PFS were 35.45 (95%CI = 34.69–36.21), 31.77 (95%CI = 29.34–34.21), and 24.32(95%CI = 21.13–27.51), respectively. For the other patients with skull base tumor, the 3-year PFS rate for F-NLR score 0, 1, 2 was 94.7%(18/19), 33.3%(5/15), and 20.0%(1/5), respectively; the mean 3-year PFS were 35.05 (95%CI = 33.25–36.86) months, 24.20 (95%CI = 18.89–29.51) months and 18.00 (95%CI = 9.50–26.50) months, respectively.

### External Validation of F-NLR Score for Predicting Prognosis

Table 4 shows the demographics and the main baseline characteristics between the training set and the validation set. There was no significant difference in age, sex, hypertension, diabetes, FIB level, NLR level, tumor size, tumor location, extent of resection, PORT, 3-year PFS rate, and F-NLR score between the two sets.

**TABLE 4:** Comparison of demographic and clinical variables in patients between training set and validation set.

Parameter	Training set year (*n* = 163)	Validation set year (*n* = 105)	*P*-value
Age	54.45 ± 11.68	52.19 ± 11.61	0.122
Sex			0.638
Male	59 (36.2%)	41 (39.0%)	
Female	104 (63.8%)	64 (61.0%)	
Hypertension			0.585
No	134 (82.2%)	89 (84.8%)	
Yes	29 (17.8%)	16 (15.2%)	
Diabetes mellitus			0.772
No	155 (95.1%)	99 (94.3%)	
Yes	8 (4.9%)	6 (5.7%)	
FIB g/L	2.97 ± 0.91	3.11 ± 1.16	0.292
NLR	2.55 ± 1.70	2.86 ± 1.81	0.167
Tumor size cm	4.93 ± 1.44	4.69 ± 1.41	0.190
Tumor location			0.613
Non-skull base	124 (76.1%)	77 (73.3%)	
Skull base	39 (23.9%)	28 (26.7%)	
Extent of resection			0.222
Simpson Grade I-II	141 (86.5%)	85 (81.0%)	
Simpson Grade III-V	22 (13.5%)	20 (19.0%)	
PORT			0.936
No	136 (83.4%)	88 (83.8%)	
Yes	27 (16.6%)	17 (16.2%)	
FPS			0.501
<3 years	45 (27.6%)	33 (31.4%)	
≥3 years	118 (72.4%)	72 (68.6%)	
F-NLR score			
0	81 (49.7%)	48 (46.7%)	0.529
1	58 (35.6%)	35 (33.3%)	
2	24 (14.7%)	21 (20.0%)	

*Values are reported as number, number(%), and mean ± standard deviation. FIB, fibrinogen; NLR, neutrophil-lymphocyte ratio; PORT, postoperative radiation therapy; PFS, progression-free survival; F-NLR, fibrinogen and neutrophil-lymphocyte ratio.*

**FIGURE 5:**
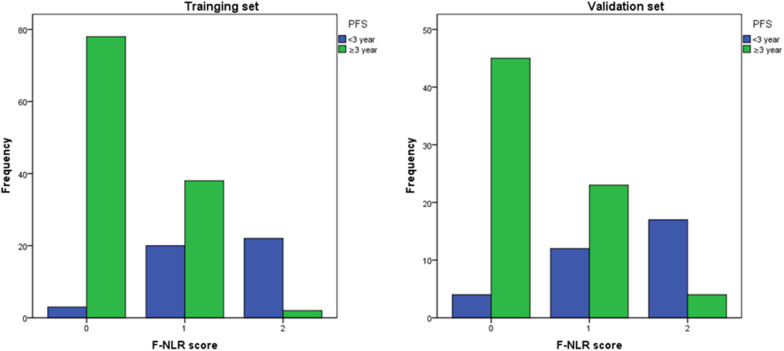
The distribution of patients with different F-NLR scores in the recurrence group and non-recurrence group was similar between the training set and the validation set.

In the validation set with 105 patients, the score of 0, 1, and 2 comprised 49 (46.7%), 35 (33.3%), and 21 (20.0%), respectively, and accounted for 45, 23, and 4 cases achieving 3-year PFS. Patients with F-NLR score of 0–2 had significantly different 3-year PFS rate both in the training set and the validation set (*P* < 0.001, *P* < 0.001, Table 5). The distribution of patients with different F-NLR scores in the two groups was similar between the training set and the validation set (Figure 5).

**TABLE 5:** The 3-year PFS rates in patients with different F-NLR scores.

	Total	PFS < 3 year (*n* = 78)	PFS ≥ 3 year (*n* = 190)	*P*-value
Training set	163 (100.0%)	45 (27.6%)	118 (72.4%)	
F-NLR score				<0.001
0	81 (49.7%)	3 (3.7%)	78 (96.3%)	
1	58 (35.6%)	20 (34.5%)	38 (65.5%)	
2	24 (14.7%)	22 (91.7%)	2 (8.3%)	
Validation set	105 (100.0%)	33 (31.4%)	72 (68.6%)	
F-NLR score				<0.001
0	49 (46.7%)	4 (8.2%)	45 (91.8%)	
1	35 (33.3%)	12 (34.3%)	23 (65.7%)	
2	21 (20.0%)	17 (81.0%)	4 (19.0%)	

*Values are reported as number, number(%). PFS, progression-free survival; F-NLR, fibrinogen and neutrophil-lymphocyte ratio.*

**FIGURE 6:**
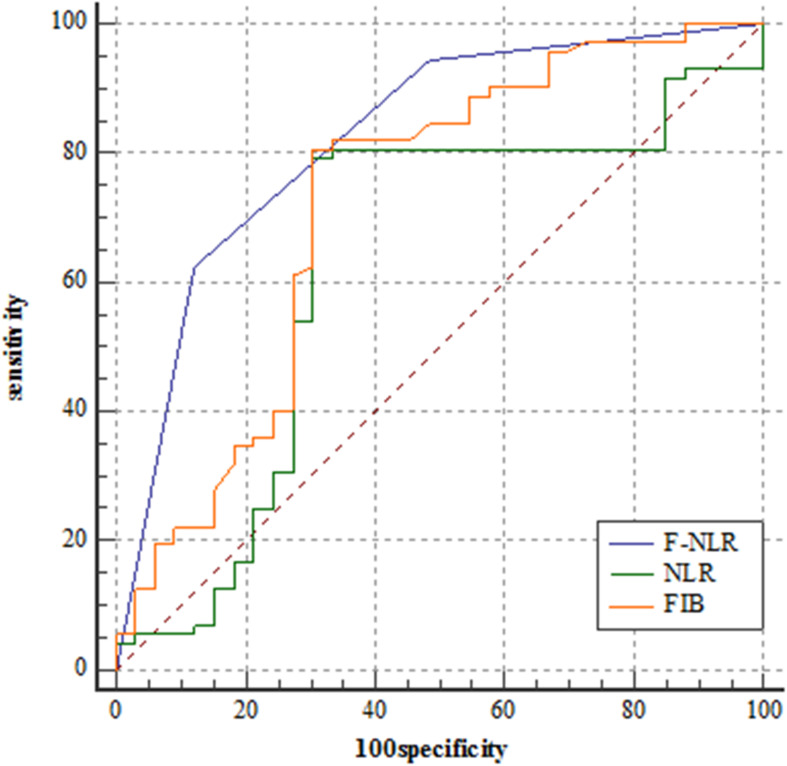
Receiver operating characteristic curve analyses comparing fibrinogen and neutrophil-lymphocyte ratio scoring system, fibrinogen level, and neutrophil-lymphocyte ratio for predicting patients reaching 3-year progression-free survival in the validation set.

Figure 6 showed the predictive performance of F-NLR scoring system compared to fibrinogen level and NLR in the validation set. Based on the cut-off value of 0, the scoring system had an AUC of 0.824 (95%CI = 0.738–0.891), a sensitivity of 62.5%, a specificity of 87.9%, and a *Youden* index of 0.504. The AUC for fibrinogen level and NLR were 0.722 (95%CI = 0.627–0.805) and 0.630 (95%CI = 0.530–0.722), respectively. DeLong’s test indicated that the AUC of F-NLR scoring system was significantly greater than those of fibrinogen level and NLR (*Z* = 2.462, *P* = 0.014; *Z* = 4.075, *P* < 0.001).

## Discussion

Tumor recurrence could increase morbidity and result in decreased survival (2). Even after gross total excision, atypical meningioma reached a high 5-year recurrence rate of 30 to 40% (2, 3). Barrett et al. reported that recurrence occurred in 30.3% of atypical meningioma patients with gross total resection at 3 years (6). Not only that, but the tumor also shows heterogeneity with variable growth rates. Hence, there is a great need to determine prognostic factors for stratifying recurrence risk of patients. Although significant progress on diagnosis and treatment of atypical meningioma has been made, there are still lots of difficulties on classifying recurrence risk of these patients. Personalized medicine based on genome sequencing may change the future of medical services. However, the procedure is invasive and may not capture the full spatial and temporal heterogeneity of the tumor. In contrast, preoperative non-invasive plasma biomarkers have enormous potential in risk stratification of tumor recurrence because they could guide surgeon to perform early and reasonable interventional therapy. Furthermore, these biomarkers could also be analyzed by conventional equipment rendering their practicability and inexpensiveness. We hypothesize that preoperative peripheral blood biomarkers could predict recurrence of atypical meningioma, allowing stratification of the extent of resection, adjuvant treatment, and tumor location.

In this study, we assessed the prognostic value of the preoperative plasma fibrinogen level and preoperative blood test in the training set with 163 atypical meningioma patients. Recurrence rate at 3 years after surgery was 29.10% in our cohort, which was similar to that reported in other studies (6). In the training set, we found a moderate predictive value of fibrinogen level (AUC = 0.786, 95%CI = 0.715–0.846, sensitivity = 77.1%, specificity = 71.1%, and *Youden* index = 0.482) in atypical meningioma relapsing at 3 year after surgery. In addition, a comparable predictive value was found in NLR (AUC = 0.743, 95%CI = 0.669–0.808, sensitivity = 87.3%, specificity = 73.3%, and *Youden* index = 0.606). Although the values of fibrinogen and NLR in predicting the 3-year PFS rate in patients with atypical meningioma were, respectively, proven, a single parameter may be not comprehensive enough. Therefore, we combined these two parameters to build a novel F-NLR scoring system and obtained a significantly elevated AUC. To the best of our knowledge, this is the first report to determine the predictive value for prognosis of F-NLR scoring system in patients with atypical meningioma. The scoring system proved its value in risk stratification of atypical meningioma recurrence at 3 years after surgery. The 3-year PFS rate decreased as the F-NLR score increased. Multivariate Cox analysis based on a multicenter retrospective study of 163 atypical meningioma patients revealed that F-NLR score was an independently related factor of 3-year PFS rate. Patients with score 1 had 12.78-fold risk of relapse than those with score 0. In addition, the risk of recurrence in patients with score 2 increased by 44.58-fold. After calculating the interaction effects, we performed subgroup analysis based on tumor location and extent of resection. Subgroup analysis based on tumor location revealed that F-NLR remained valuable in predicting 3-year PFS rate (*P* < 0.001). Kaplan-Meier curve analysis revealed that for patients with Simpson grade I-II and Simpson grade III-V, different F-NLR scores were both associated with significantly different 3-year PFS rates (*P* < 0.001, *P* = 0.045). This result strengthened the idea that F-NLR scoring system and 3-year PFS were closely related. In addition, the good predictive performance of F-NLR scoring system was also demonstrated in the validation set with 105 patients (AUC = 0.824, 95%CI = 0.738–0.891, sensitivity = 62.5%, specificity = 87.9%, and *Youden* index = 0.504). As the similar baseline characteristics and F-NLR score distribution between the training set and the validation set, we had reason to believe the predictive value of F-NLR scoring system in prognosis of patients with atypical meningioma.

Accumulating evidence demonstrates the important pathophysiological role of activation of coagulation cascade in tumor progression. Fibrinogen, a key component in the coagulation system, has been confirmed as an important regulator of systemic inflammatory response and cancer development in various types of tumor. Many studies reported the association between hyperfibrinogenemia and invasiveness of malignancy (9–13). Plasma fibrinogen level was also confirmed to be significantly lower in WHO grade I meningioma compared to that of glioblastomas and metastases (21). In meningioma of different grades, immunohistochemical analysis revealed that fibrinogen staining scores were significantly elevated from grade I to grade III. These pathological or clinical evidences provide a theoretical basis for application of fibrinogen level in predicting prognosis in patients with atypical meningioma.

However, the mechanism of regulation remains unclear, with the following possible explanations. First, a “web” built by fibrinogen in the extracellular matrix could promote cell adhesion, migration, and invasion of tumor (22, 23). Second, the physical barrier formed by platelet-fibrin deposition surrounding tumor cells could prevent them from the killing contact of NK cells (24). In atypical meningioma, fibrinogen was observed to surround tumor cells in a fibrillary pattern (25). Third, as an acute-phase reactant released in malignancy and systemic inflammation, fibrinogen could be synthesized by tumor cells and promoted to be released by interleukin-6 (26). In turn, the released fibrinogen promotes tumor cell proliferation by the combined effects with vascular endothelial growth factor and fibroblast growth factor-2 (27, 28). In our study, patients with higher preoperative fibrinogen level had lower 3-year PFS rate than the others. Hence, fibrinogen could be a reliable predictor for the prognosis of atypical meningioma.

In recent years, systemic inflammation has attracted significant attention in tumor occurrence and progression. The predictive value of NLR, a representative indicator of tumor-related inflammation, for intracranial tumor prognosis has been confirmed (12, 29). Neutrophils are known for playing a positive role in tumor growth and angiogenesis (30). Conversely, lymphocytes make a significant contribution in inhibiting tumor proliferation (31). Therefore, the neutrophil-lymphocyte ratio could enhance the efficacy for predicting recurrence risk in malignant tumor. For meningioma, the value of preoperative blood test in predicting tumor grades has been confirmed (32). Our study revealed that atypical meningioma patients with high NLR had lower 3-year PFS rate than those with low NLR. Therefore, NLR could be used as a predictor to evaluate the recurrence risk of atypical meningioma.

In our study, we also evaluated the predictive value of several other clinic-pathological factors on the tumor recurrence of atypical meningioma. In variable types of tumor, elevated Ki-67 index and mitotic level played a key role in aggressiveness and tendency to recurrence (33, 34). Brain invasion and high mitotic index has been shown to be linked with higher risk of recurrence in atypical meningioma (5). Similarly, as a cellular biomarker of proliferation, Ki-67 index has been successfully applied in predicting local recurrence of an atypical meningioma cohort following gross total resection (6). Unfortunately, our study did not find the association between Ki-67 index and 3-year PFS in patients with atypical meningioma. The relationship between brain invasion, mitotic index and 3-year PFS was also not statistically significant in univariate analysis. That may be due to insufficient sample size. Extent of resection was highly influential on atypical meningioma recurrence which has been confirmed in many studies (7, 35, 36). Thus, it is recommended that atypical meningioma should be completely resected based on Simpson grade. In our study, we divided the patients into Simpson grade I-II group and Simpson III-V group due to small number of cases in Simpson grade IV-V. Multivariate logistic regression analysis showed the strong relation between extent of resection and 3-year PFS. The Simpson grade III-V group had 2.59-fold risk of relapse than the other group in the univariate Cox hazard regression analysis. However, after multivariate analysis, extent of resection lost statistical significance possibly due to the insufficient sample in the training set. Actually, we have also created a rating scale incorporating all those independent prognostic factors that resulted significant at the multivariate analysis before updating the manuscript. But we found the predictive value of that rating scale was similar to our F-NLR scoring system (AUC = 0.895, 95%CI = 0.838–0.938 vs. AUC = 0.872, 95%CI = 0.811–0.919; *Z* = 1.140, *P* = 0.254). And the rating scale incorporating all the independent prognostic factors was not convenient in the clinical application. Our F-NLR scoring system incorporating two preoperative blood test parameters was relatively objective and easy to access. Therefore, we retained the F-NLR scoring system to predict prognosis of patients in our study.

Many potential factors contribute to the formation of peritumoral edema with complex mechanism. Peritumoral edema also demonstrated significant link with histopathological grade and aggressive growth of tumor in some studies (8, 37). However, others also showed negative investigations on the uncertain relationship (38, 39). Similarly, our study showed that peritumoral edema of atypical meningioma was not correlated with tumor recurrence. On the other hand, prior studies have demonstrated that larger tumor size was correlated with high tumor proliferative potential (35, 40). Our findings confirmed the higher risk of recurrence in larger tumor in both univariate and multivariate analyses. Tumor location could affect the difficulty of surgery and the extent of resection. Atypical meningioma in skull base is more difficult to be completely resected, which contributes to the tendency of recurrence. In our study, tumor locating in skull base was confirmed as an independent risk factor for 3-year PFS in multivariate analysis. Fukushima et al. reported that skull base location was independently associated with tumor recurrence in patients following Simpson Grade IV resection (41). Another study also validated the higher recurrence rate of skull base tumor in a Grade I-V cohort (42). These studies shed light on our findings and our study also extended theirs.

Given the potential toxicities of upfront PORT, there is no consensus on the necessity for patients with atypical meningioma taking it as a conventional therapy. The European Association of Neuro Oncology guidelines recommend that adjuvant radiation therapy should be considered in tumor with incomplete resection (43). On the other hand, a Phase III trial conducted by the European Organization for Research and Treatment of Cancer is currently ongoing to clarify the value of PORT in patients after complete resection (44). However, prior studies were contradictory on whether PORT decreases the risk of recurrence in atypical meningioma patients. Some studies affirmed the efficacy of PORT while others showed a non-significant association between PORT and tumor recurrence (36, 45, 46). In our cohort, we did not find the value of PORT in decreasing the risk of tumor recurrence.

Several limitations existed in our study. First, although the acquired data were from multiple centers, the retrospective design of study suffered potential selection bias. Second, other molecular markers were not included in our study because of incomplete immunohistochemical analysis in early cases. Third, the minimum duration of follow-up was not long enough to evaluate further PFS rate and overall survival of patients. Therefore, a multicenter prospective study with more thorough and comprehensive data should be carried out to validate the reliability of F-NLR scoring system in patients with atypical meningioma again.

## Conclusion

Our study confirmed the correlation and clinical significance of preoperative F-NLR scoring system with 3-year PFS of patients with atypical meningioma. Extent of resection, tumor size, and tumor location also showed their association with 3-year PFS. The F-NLR scoring system could serve as a useful tool for predicting prognosis of patients with atypical meningioma. A prospective and large-scale study is required to validate our findings.

## Data Availability Statement

The raw data supporting the conclusions of this article will be made available by the authors, without undue reservation.

## Ethics Statement

The studies involving human participants were reviewed and approved by the Ethics Committee of The First Affiliated Hospital of Fujian Medical University, The Affiliated Hospital of Putian University, and the Henan Provincial People’s Hospital. The patients/participants provided their written informed consent to participate in this study.

## Author Contributions

CD, DK, and JG performed and designed the experiments, analyzed and interpreted the data, and wrote the manuscript. XC, GW, JZ, GZ, YL, and ZL collected and interpreted the data. All authors were involved in drafting the article and reviewed and approved the final manuscript.

## Conflict of Interest

The authors declare that the research was conducted in the absence of any commercial or financial relationships that could be construed as a potential conflict of interest.
